# Au modified PrFeO_3_ with hollow tubular structure can be efficient sensing material for H_2_S detection

**DOI:** 10.3389/fbioe.2022.969870

**Published:** 2022-08-24

**Authors:** Heng Zhang, Jing Xiao, Jun Chen, Lian Zhang, Yi Zhang, Pan Jin

**Affiliations:** ^1^ College of Physics and Electronic Engineering, Taishan University, Taian, Shandong, China; ^2^ Health Science Center, Yangtze University, Jingzhou, Hubei, China; ^3^ Collaborative Innovation Centre of Regenerative Medicine and Medical BioResource Development and Application Co-constructed by the Province and Ministry, Guangxi Medical University, Nanning, Guangxi, China

**Keywords:** gas sensor, Au-PrFeO_3_, H_2_S, moss, gas-sensing

## Abstract

The H_2_S concentration in exhaled breath increases marginally with the progress of periodontal disease, and H_2_S is considered to be one of the most important gases related to meat and seafood decomposition; however, the concentration of H_2_S is low and difficult to detect in such scenarios. In this study, Au–PrFeO_3_ nanocrystalline powders with high specific surface areas and porosities were prepared using an electrospinning method. Our experimental results show that loading Au on the material provides an effective way to increase its gas sensitivity. Au doping can decrease the material’s resistance by adjusting its energy band, allowing more oxygen ions to be adsorbed onto the material’s surface due to a spillover effect. Compared with pure PrFeO_3_, the response of 3 wt% Au–PrFeO_3_ is improved by more than 10 times, and the response time is more than 10 s shorter. In addition, the concentration of H_2_S due to the decomposition of shrimp was detected using the designed gas sensor, where the error was less than 15%, compared with that obtained using a GC-MS method. This study fully demonstrates the potential of Au–PrFeO_3_ for H_2_S concentration detection.

## Introduction

H_2_S is a colorless, highly toxic, and acidic gas. It has a particular rotten egg smell, and even low concentrations of H_2_S can impair the human sense of smell. In high concentrations, it has no smell (as high concentrations paralyze the olfactory nerve). In addition, H_2_S is flammable and is typically considered a dangerous gas (Ethiraj et al., 2015; Wu et al., 2019; Kumar et al., 2021; Liu et al., 2021; Priya et al., 2021; Zheng et al., 2021; Zuo et al., 2021; Li et al., 2022a). H_2_S gas is released during the breakdown of food, and is also responsible for the bad breath caused by periodontitis (Chen et al., 2017; Wu et al., 2019; Hsu et al., 2021; Lopez et al., 2021): about 0.195 ppm H_2_S can be detected in the exhaled breath of periodontitis patients, while 0.105 ppm is a typical concentration in the exhaled breath of healthy individuals (Yaegaki and Sanada, 1992). Using the nose as a means of detecting H_2_S can be fatal. Therefore, the timely detection of very low concentrations of H_2_S gas is very necessary and important.

In recent years, the use of MOS (metal oxide semiconductor) gas sensors to detect the concentration of target gases has become increasingly popular, such as smoke sensors in hotels, natural gas alarms in homes, and so on. It has been reported that some MOSs, as gas-sensing materials, show excellent response to gases, such as LaFeO_3_ (Xiangfeng and Siciliano, 2003; Song et al., 2014; Jaouali et al., 2018; Ma et al., 2021), SmFeO_3_ (Tomoda et al., 2004; Hosoya et al., 2005; Huang et al., 2018; Han et al., 2020), PrFeO_3_ (Ma et al., 2018), HoFeO_3_ (Song et al., 2020), NdFeO_3_ (Sheng et al., 2022), YCoO_3_ (Addabbo et al., 2015), BaSnO_3_ (Cerdà et al., 2002), ZnSnO_3_ (Yin et al., 2020), and YMnO_3_ (Balamurugan and Lee, 2015). For H_2_S, commonly used gas-sensing materials include Pt–ZnO (Zhou et al., 2022), Pd–ZnO (Bae et al., 2022), CuO/SnO_2_ (Fan et al., 2019), Pt–WO_3_ (Yao et al., 2022), WO_3_ (Wang et al., 2018; Akamatsu et al., 2021; Li et al., 2022b), Pt–Fe_2_O_3_ (Guo et al., 2018), CuO/CuFe_2_O_4_(Lim et al., 2021), Ag–SnO_2_ (Senapati and Sahu, 2020), LaFeO_3_ (Xiangfeng and Siciliano, 2003), YMnO_3_ (Balamurugan and Lee, 2015), and Sn–NiO (Gao et al., 2017), among others. MOSs—especially ABO_3_ perovskite materials—have the unique advantages of large specific surface area and abundant active sites, which can promote the diffusion path and increase the adsorption of target gas molecules, thus enhancing the sensing ability. There are other ways to detect a target gas, such as Tamm plasmon resonance (Mehaney et al., 2021) and photonic crystal (Ahmed et al., 2021; Ameen et al., 2021; Alrowaili et al., 2022). In particular, these two methods have high accuracy in detecting the gases in exhaled breath, and have good development prospect.

In recent years, sensors based on Graphene and MWCNT have been widely reported, especially for gases in exhaled breath or aromatic gases (Behi et al., 2020, 2022; Thamri et al., 2021). Such sensors display high response and selectivity to target gases; moreover, they have good development prospect due to their low preparation costs.

The aim of this study is to obtain a gas-sensing material with high response, high selectivity, low detection limit, and high long-term stability. PrFeO_3_ with different Au doping levels was synthesized using an electrospinning method and sintered at 800°C. It has high specific surface area and high porosity, which are two important factors for improving the gas response of gas-sensing materials. Compared with PrFeO_3_, Au–PrFeO_3_ shows a higher response and high selectivity for H_2_S. In addition, Au doping, as a catalyst, can greatly enhance the surface activity of gas-sensing materials, thus shortening the response-recovery time. Finally, the H_2_S concentration in the air around shrimp is detected using the gas sensor designed in this study, which was compared with the data obtained by GC-MS, showing that the error was within 15%. The experimental results prove that Au doping can greatly improve the response of PrFeO_3_ to H_2_S gas, providing a feasible and effective way to detect H_2_S gas using a gas sensor.

## Materials and methods

### Preparation of nanocrystalline Au–PrFeO_3_

**FIGURE 1:**
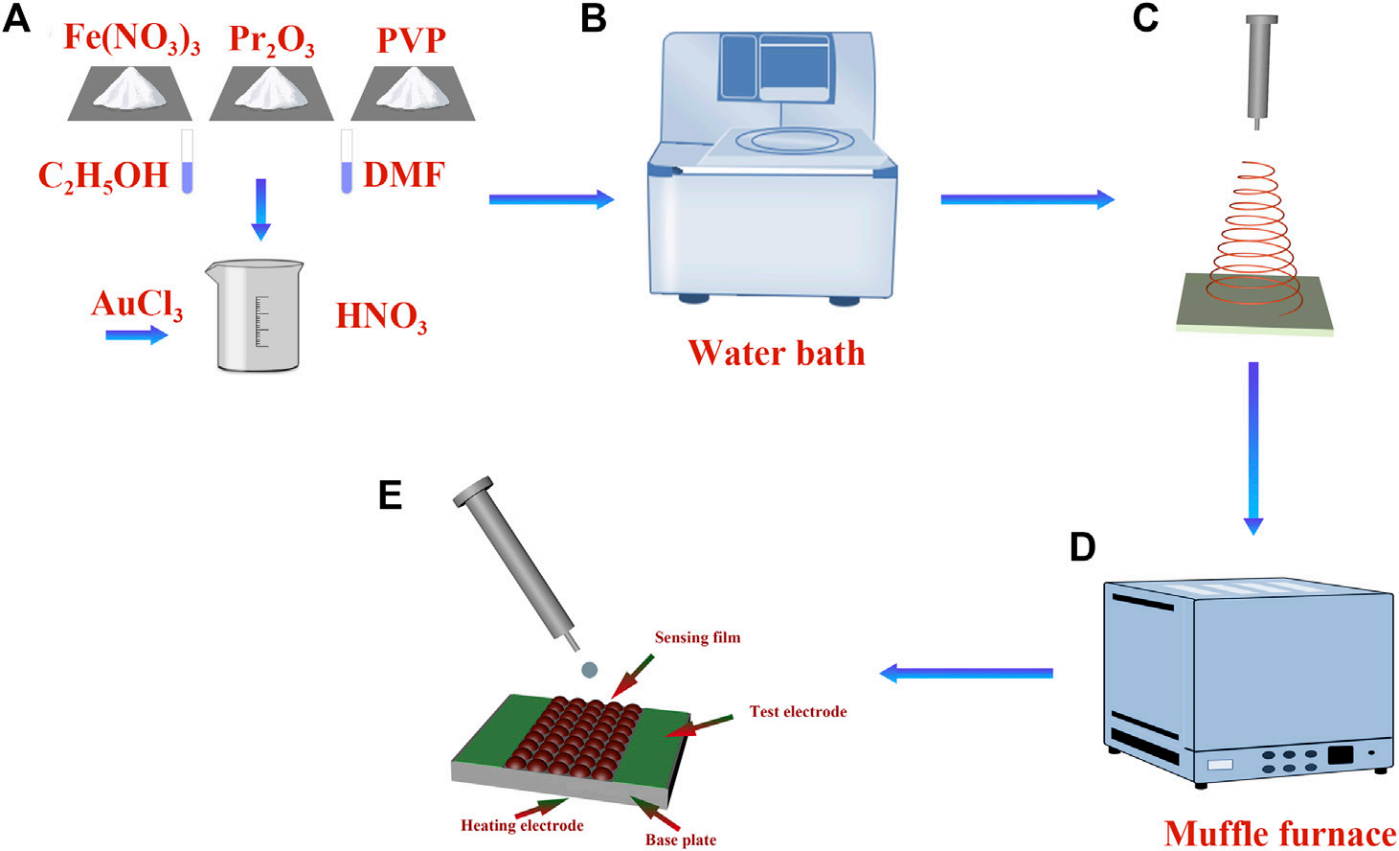
**(A–D)** The flow chart of Au-PrFeO_3_ preparation; **(E)** The gas sensor structure diagram.

First, samarium oxide, ferric nitrate, palladium chloride, DMF (99.5%), PVP (Mw = 1,300,000), C_2_H_5_OH (99.7%), and HNO3 in a stoichiometric ratio were weighed and mixed in deionized water (Figure 1A). The mixed solution was heated in the water bath at 60°C with magnetic stirring until it became transparent, in order to obtain the electrospinning precursor solution (Figure 1B). Then, the as-prepared precursor solution was transferred into a 10 mL syringe. The voltage was maintained at 12 kV during the spinning process, the distance between needle tip and the collector was about 20 cm, and the injection rate of the syringe was 0.4 mL/h (Figure 1C). The obtained nanofibers were sintered at 800°C for 6 h in a muffle furnace (Figure 1D). Finally, the X (0, 1, 3, 5) wt% Au–PrFeO_3_ powder was obtained.

### Fabrication and measurement of sensor

The Au–PrFeO_3_ powder was mixed with deionized water to make a paste, which was then placed on gas-sensing film (Figure 1E). The as-prepared plane electrode plate was aged on an aging platform for 48 h, in order to dry it out. At this time, a qualified sensor was ready. The front side of the electrode plate has two electrodes, which are used to detect the resistance value of the gas-sensing material. On the back of electrode plate is a heating plate, which enables the gas-sensing material to reach a higher operating temperature.

### Ready-made sensor

The gas sensor structure diagram is shown in Figure 1E. The Au–PrFeO_3_ is coated onto the sensing film. The $V_{C}$ is the supply voltage, which was kept constant at 5 V. The $R_{g}$ is calculated by the following formula:

$$\begin{array}{c} R_{g}=\;\frac{U}{I}=V_{l}/R_{l}\; \end{array}$$ (1)

The gas-sensing response, *S,* is defined as $R_{g}/R_{a}$ , where $R_{a}$ is the resistance of the sensor in air and $R_{g}$ is that when in the tested gas. The response time is defined as the time taken to attain 90% of the maximum value in ascending phase, while the recovery time is the time taken to regain 10% of the base value in the descent phase. For the experimental environment, the RH was 20% and the temperature was 20°C.

### Gas concentration control

The whole experiment was carried out in a closed glass chamber, into which H_2_S gas was injected with a microinjector. The injection amount of H_2_S liquid was calculated as follows (Deng et al., 2013):

$$\begin{array}{c} V_{liquid}=\frac{V_{s}C_{gas}M}{22.4\rho d} \end{array}$$ (2)

where $V_{liquid}$ is the volume of the injected H_2_S liquid; $V_{s}$ is the volume of the test chamber; $C_{gas}$ (ppm) is the concentration of the target gas; and M , ρ, and d are the molecular weight, density, and purity of the injected liquid, respectively. When a gas with PPM concentration is obtained, the gas with PPB concentration can be obtained by diluting it ten times using a microinjector.

## Results

### Material characterization

**FIGURE 2:**
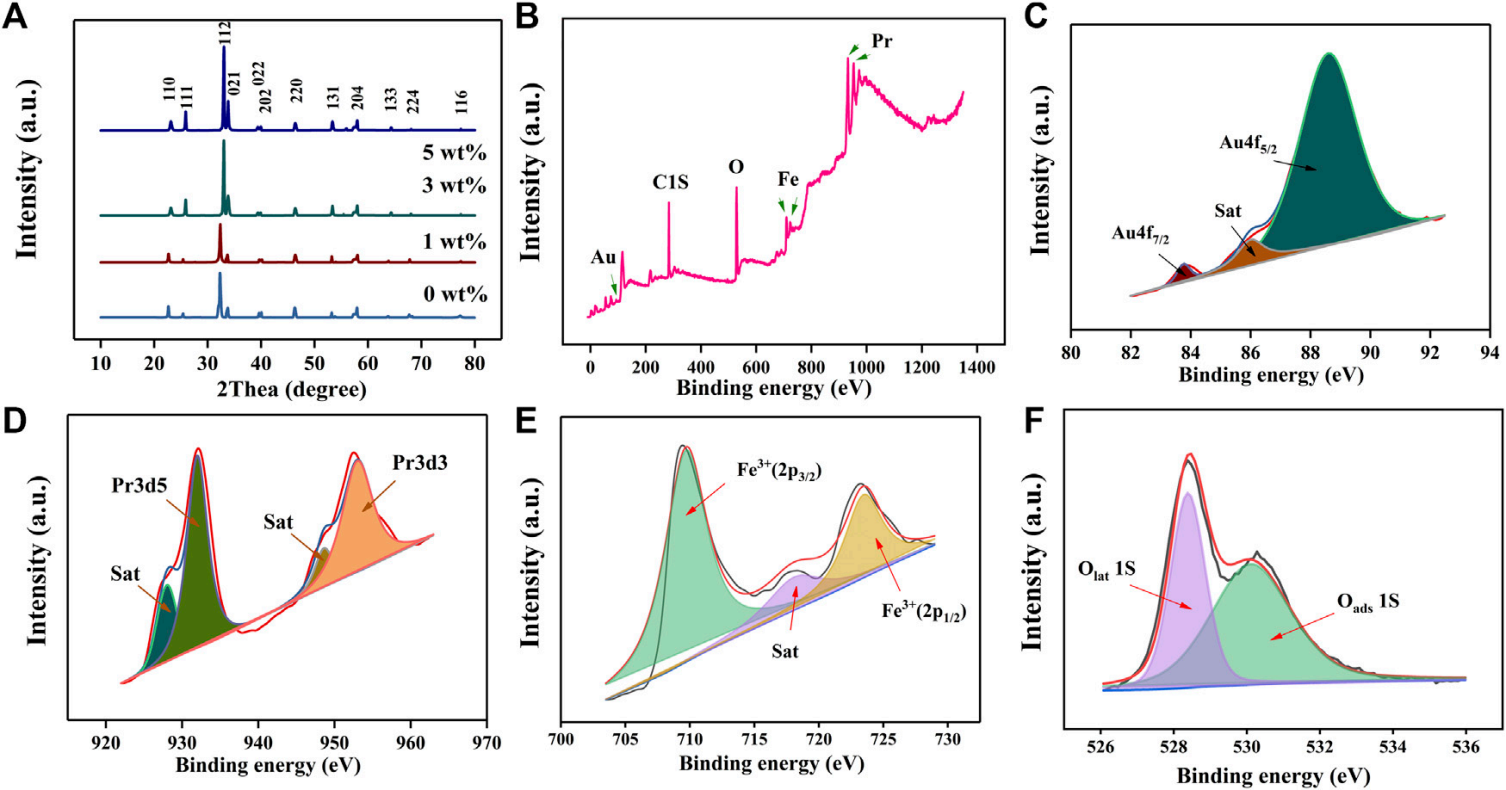
**(A)** The XRD pattern of X (0–5) wt% Au-PrFeO_3_; **(B)** The XPS survey of 3 wt% Au-PrFeO_3_; **(C–F)** The fine spectra of XPS analysis for each element (Au, Pr, Fe, O).

Figure 2A shows the X-ray diffraction analysis (XRD; Bruker D8 ADVANCE with the CuKα amount of 1.5405 Å at 40 kV and 40 mA) results of X (0, 1, 3, 5) wt% Au–PrFeO_3_. Compared with the standard card (PDF card: 37-1493), it shows a single-phase. The average particle size can be calculated using the Scherrer method. The Scherrer equation is as follows:

$$\begin{array}{c} D=\;\frac{k\lambda }{\beta cos\theta } \end{array}$$ (3)

where λ is the X-ray wavelength, β is the integral width of diffraction peaks, and θ is the Bragg diffraction angle. The average particle size of 3 wt% Au–PrFeO_3_ is about 73.8 nm. Due to the low Au doping amount, its characteristic peak could not be reflected in the XRD pattern; therefore, X-ray Photoelectron Spectroscopy (XPS; Thermo Scientific™ K-Alpha™^+^ spectrometer equipped with a monochromatic Al Kα X-ray source at 1486.6 eV operating at 100 W) was performed on 3 wt% Au–PrFeO_3_ to confirm the presence of Au. As can be seen from Figure 2B, the Au element was doped in the material. Figures 2C–F show the fine spectra obtained by XPS analysis for each element. In Figure 2C, the peaks located at about 84.0 and 88.3 eV can be assigned to Au 4f_7/2_ and Au 4f_5/2_; in Figure 2D, the peaks located at about 932.2 and 953.1 eV can be assigned to Pr 3d5 and 3d3; in Figure 2E, the peaks located at about 708.6 and 724.1 eV can be assigned to 2p_3/2_ and 2p_1/2_ of Fe^3+^; and, in Figure 2F, the peaks located at about 528.6 and 530.8 eV can be assigned to lattice O1s and adsorbed O1s.

**FIGURE 3:**
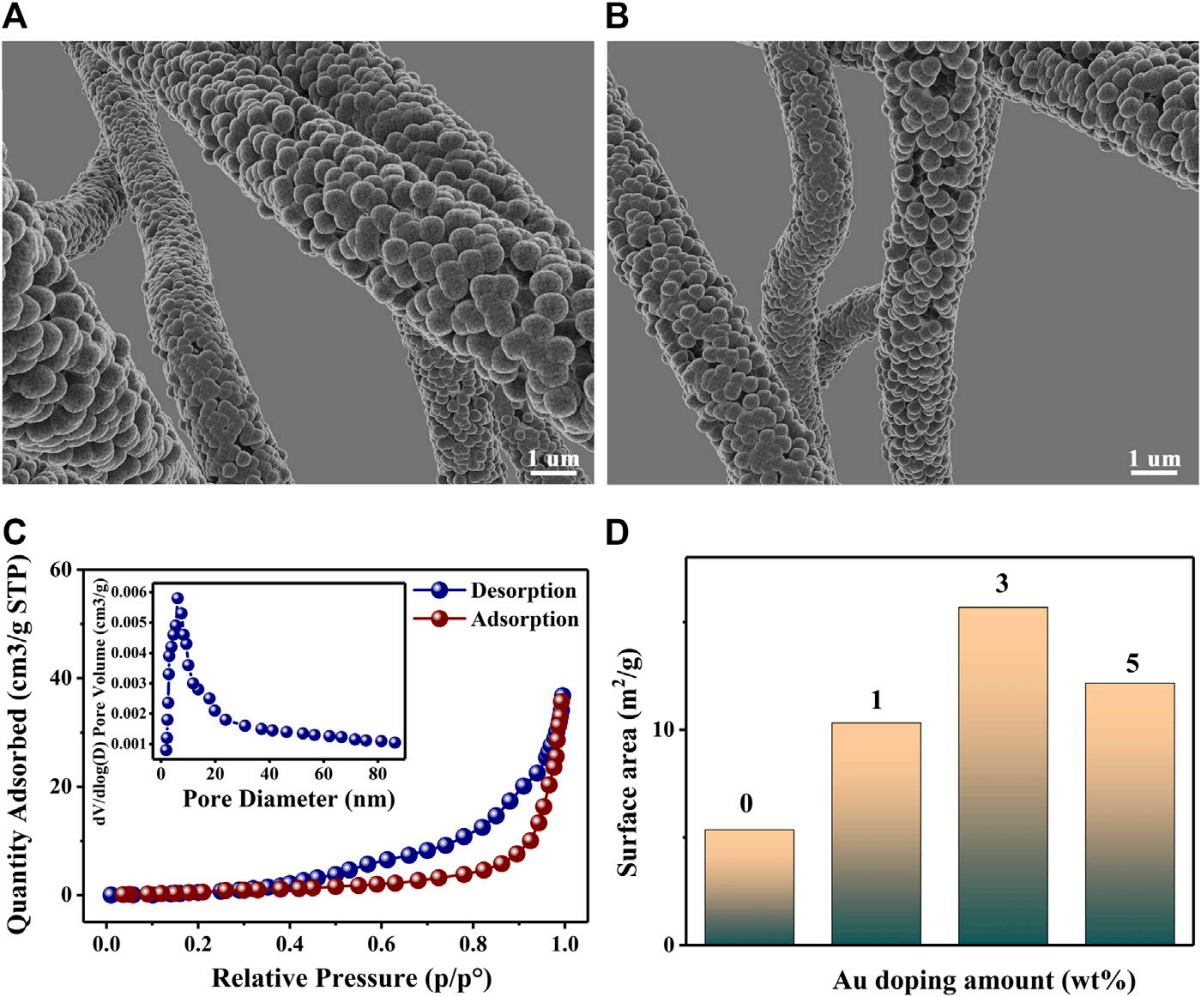
**(A,B)** The SEM of 3 wt% Au-PrFeO_3_; **(C)** N_2_ adsorption–desorption isotherms and pore size distributions (the inset) for Au-PrFeO_3_ nanocomposite; **(D)** The surface area of PrFeO_3_ with different amount of Au doping.

Figures 3A,B shows the Scanning Electron Microscope (SEM; Japan HITACHI SU8010, 8.0 kV) images of 3 wt% Au–PrFeO_3_ under different magnifications (PrFeO_3_ was synthesized by a sol-gel method and sintered at 800°C; Supplementary Figure S1). The pure PrFeO_3_ presented a common perovskite structure, while 3 wt% Au–PrFeO_3_ presented a nanotube-like microstructure. In the material preparation stage, after sintering, the surface of the material becomes rough and the nanotubes become hollow as the PVP decomposes at high temperature.

In order to understand which microstructure provides more favorable properties to the gas-sensing material, it is necessary to figure out which structure has higher specific surface area and porosity. The specific surface area and porosity of the 3 wt% Au–PrFeO_3_ hollow nanofibers were further analyzed by nitrogen adsorption–desorption analysis. Figure 3C shows the BET curves for 3 wt% Au–PrFeO_3_ and the corresponding Barrett–Joyner–Halenda (BJH) pore size distribution (inset). The specific surface area of 3 wt% Au–PrFeO_3_ was 23.67 m^2^/g and the average pore size was 10.2 nm. The specific surface areas of PrFeO_3_ with different amounts of Au element doping are shown in Figure 3D. It can be seen that, when the doping amount of Au element was 3 wt%, the composite powder presented the largest specific surface area. This occurred as Au doping can inhibit the growth of MOS grains (the smaller the grain size, the larger the specific surface area); however, when the Au doping amount is too high, the particles will appear in a small range of agglomeration, and the specific surface area of the material will decreased. Considering the sensing properties of materials, the specific surface area is an important factor. A high specific surface area can provide more adsorption sites, which can enhance the reactions between the sensing material and gas molecules, leading to a high response to the test gas.

### Gas sensing performance

**FIGURE 4:**
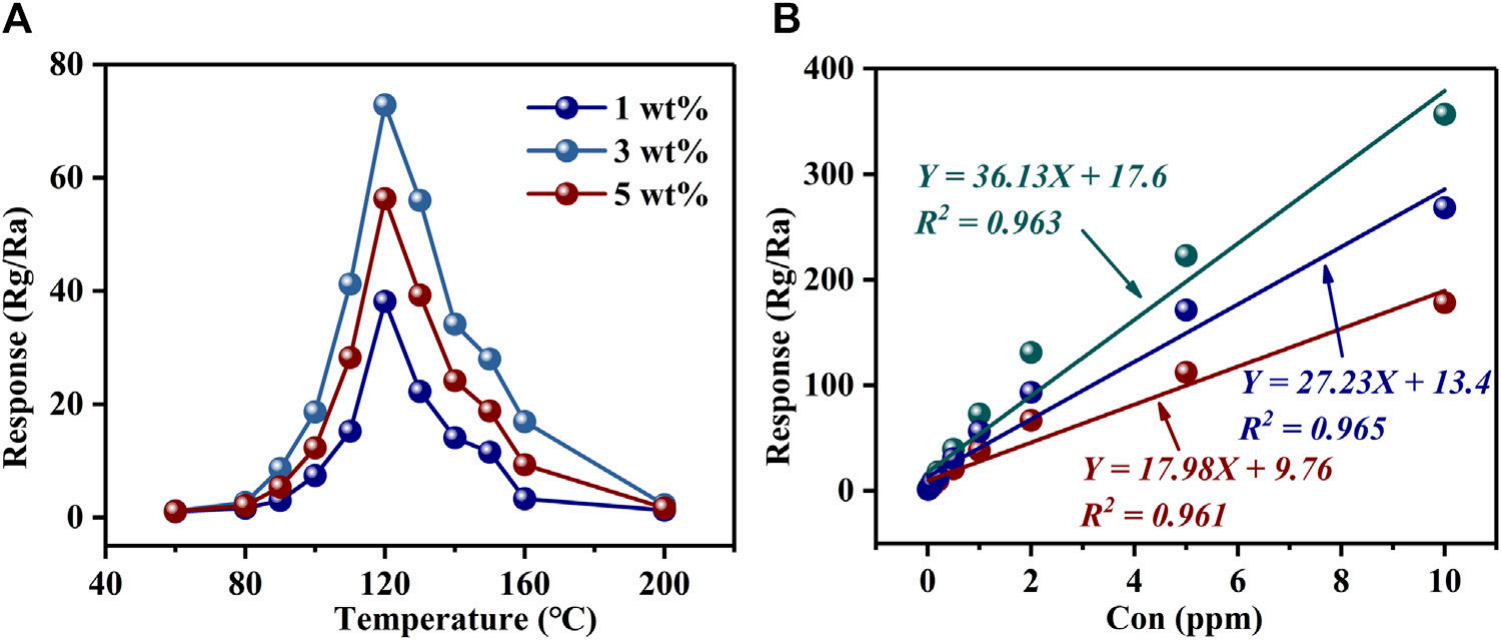
**(A)** The response of PrFeO_3_ with Au doping; **(B)** The relationship between the response of Au-PrFeO_3_ and multiple H_2_S concentration.

Figure 4A and Supplementary Figure S2 show the response curves of PrFeO_3_ with different amount of Au doping to 1 ppm H_2_S at various operating temperatures. For all samples, the highest responses were obtained at 120°C. The highest responses to 1 ppm H_2_S were 6.93 (0 wt% Au), 38.16 (1 wt% Au), 72.86 (3 wt% Au), and 56.29 (5 wt% Au). It can be seen that the response was more than 10 times higher when using the best Au-doped sample, compared with the pure sample. Moreover, Table 1 shows the H_2_S sensing properties of some typical gas-sensing materials for reference. By comparison, 3 wt% Au–PrFeO_3_ exhibited an extremely high response value while ensuring a short response–recovery time.

**TABLE 1:** The H_2_S sensing performance of materials in the literature and this work.

Materials	T (°C)	S (ppm)	T_res_/T_rec_ (s)	Detection limit (ppm)	Ref
rGO/WO3	300	22.9 (100)	23/75	1	Mehta et al. (2021)
Fe2O3/MoSe2	25	42.5 (10)	50/53	1	Pan et al. (2021)
WO3/Bi2W2O9	92	84.18 (100)	2/582	0.01	Zhang et al. (2020)
CuO/WO3-x	99	171.5 (10)	45/60	0.1	Peng et al. (2020)
Pt–Co3O4@NiO	200	250.0 (100)	213/135	20	Wang et al. (2021)
Pt–WO3	200	1638.2 (10)	42/37	0.005	Yao et al. (2022)
3 wt% Au–PrFeO3	120	72.86 (1)	28/18	0.01	This work

The relationship between the material’s sensitivity and the gas concentration is very important, and a good fitting relationship can be used to predict the response value at a given gas concentration. Figure 4B and Supplementary Figure S3 show the relationship between the response of Au–PrFeO_3_ and multiple H_2_S concentrations. It can be seen that, for both undoped and Au–doped PrFeO_3_, the response had a good linear relationship with the gas concentration, with all $R^{2}$ values greater than 96%. Additionally, the response values of Au–doped PrFeO_3_ to H_2_S are given in Table 2. It can be seen that the detection limit of pure PrFeO_3_ was 50 ppb; meanwhile, after Au doping, the Au–PrFeO_3_ could detect a much lower concentration (10 ppb) of H_2_S.

**TABLE 2:** The response of Au–PrFeO_3_ to H_2_S gas.

Con (ppm) Doping amount	0.01	0.02	0.05	0.1	0.2	0.5	1	Detection limit (ppm)
0 wt%			1.21	1.62	2.38	4.06	6.93	0.05
1 wt%	1.18	1.58	2.94	5.45	9.93	20.85	38.16	0.01
3 wt%	1.26	2.43	4.78	9.32	17.9	39.38	72.86	0.01
5 wt%	1.21	1.98	3.68	7.56	13.56	30.59	56.29	0.01

**FIGURE 5:**
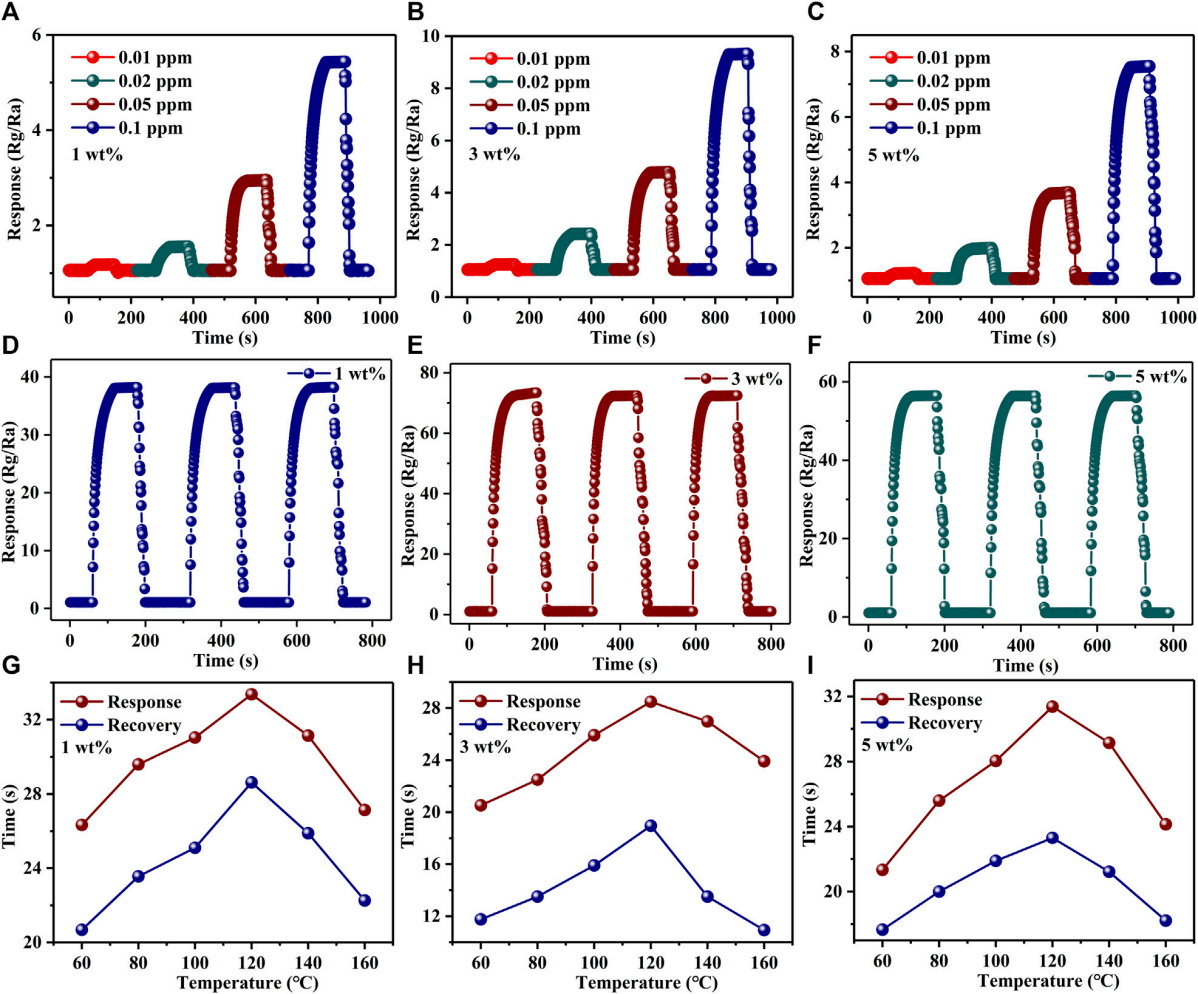
**(A–C)** The repeatability of responses of PrFeO_3_ with Au doping to different concentration of H_2_S gas; **(D–F)**; The repeatability of responses of PrFeO_3_ with Au doping to 1 ppm H_2_S gas; **(G–I)** The response-recovery time of Au-PrFeO_3_ to 1 ppm H_2_S.

Repeatability is another important property that determines whether a gas-sensing material is excellent or not. For Au–PrFeO_3_, the repeatability of responses to different concentrations of H_2_S gas are shown in Figures 5A–C and Supplementary Figure S4. The repeated processes were carried out as follows: when the resistance value of the gas-sensing material had stabilized, the H_2_S gas was injected into the reaction chamber, and the resistance of the material increased immediately. After a period of time, the resistance stabilized, following which the H_2_S gas is removed and the resistance of the material decreased immediately, restoring it to the initial state. It can be seen that, for H_2_S gas at different concentrations, the resistance of the gas-sensing material could be restored to the initial value every time after the H_2_S gas was removed, indicating that the material has excellent repeatability. Additionally, the response of all samples changed upon exposure to 1 ppm H_2_S gas, as shown in Figures 5D–F. It can be seen that the gas response of samples had no obvious change after a 3-cycle response–recovery test, indicating the high operating stability of the designed Au–PrFeO_3_ sensor. Additionally, the gas-sensing reproducibility of Au-PrFeO_3_ is about 38.16 ± 4% (1 wt%), 72.86 ± 2% (3 wt%), 56.29 ± 3.6% (5 wt%).

The response–recovery time of all samples differed at different operating temperatures, indicating that the operating temperature affects the chemical reaction on the material’s surface. The response–recovery times of all samples are shown in Supplementary Table S1, Figures 5G–I, and Supplementary Figure S5. It can be seen that the response–recovery time increased with the operating temperature up to 120°C; then, after 120°C, the response–recovery time decreased with any further increase in the operating temperature. This may be due to the fact that, before the optimum operating temperature, the adsorption rate of gas molecules is higher than the desorption rate, and the number of oxygen ions and H_2_S gas molecules adsorbed on the material’s surface are increased, leading to an increased reaction time. With an increase in the operating temperature, the adsorption and desorption rates are balanced at the optimum operating temperature, and the number of H_2_S gas molecules and adsorbed oxygen ions on the material’s surface reach a maximum. At this operating temperature, the reaction time also reaches its maximum. With a further increase in operating temperature, the desorption rate of gas molecules is higher than the adsorption rate, the reaction reactants become less, and the reaction time is shortened. In addition, Au doping can increase the surface activity of the material and improve the reaction rate; therefore, the response–recovery time of Au–PrFeO_3_ was shorter than that of pure PrFeO_3_.

**FIGURE 6:**
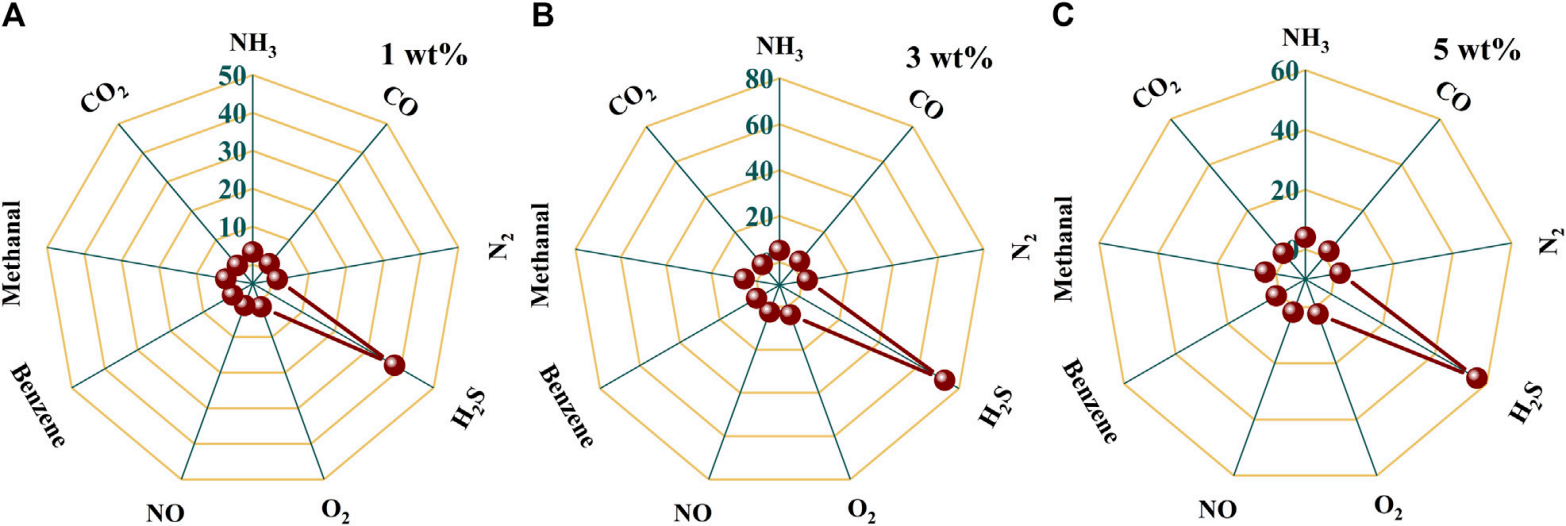
**(A–C)** The selectivity comparison of Au-PrFeO_3_ to 1 ppm H_2_S and several other common gases.

In practical application, it is very common to detect a certain gas in a mixture, such as H_2_S gas in an individual’s exhaled breath. Therefore, the selectivity of a gas-sensing material to a certain gas determines its practical application value. The selectivity comparison of Au–PrFeO_3_ to 1 ppm H_2_S and several other common gases in a person’s exhaled breath is shown in Figures 6A–C and Supplementary Figure S6. It can be seen that, compared with other gases, Au–PrFeO_3_ presented high selectivity for H_2_S gas. In particular, for N_2_, O_2_, NO, CO_2_, CO, and other common gases present in exhaled breath, the response was negligible, such that the H_2_S in the exhaled breath can be detected more accurately.

**FIGURE 7:**
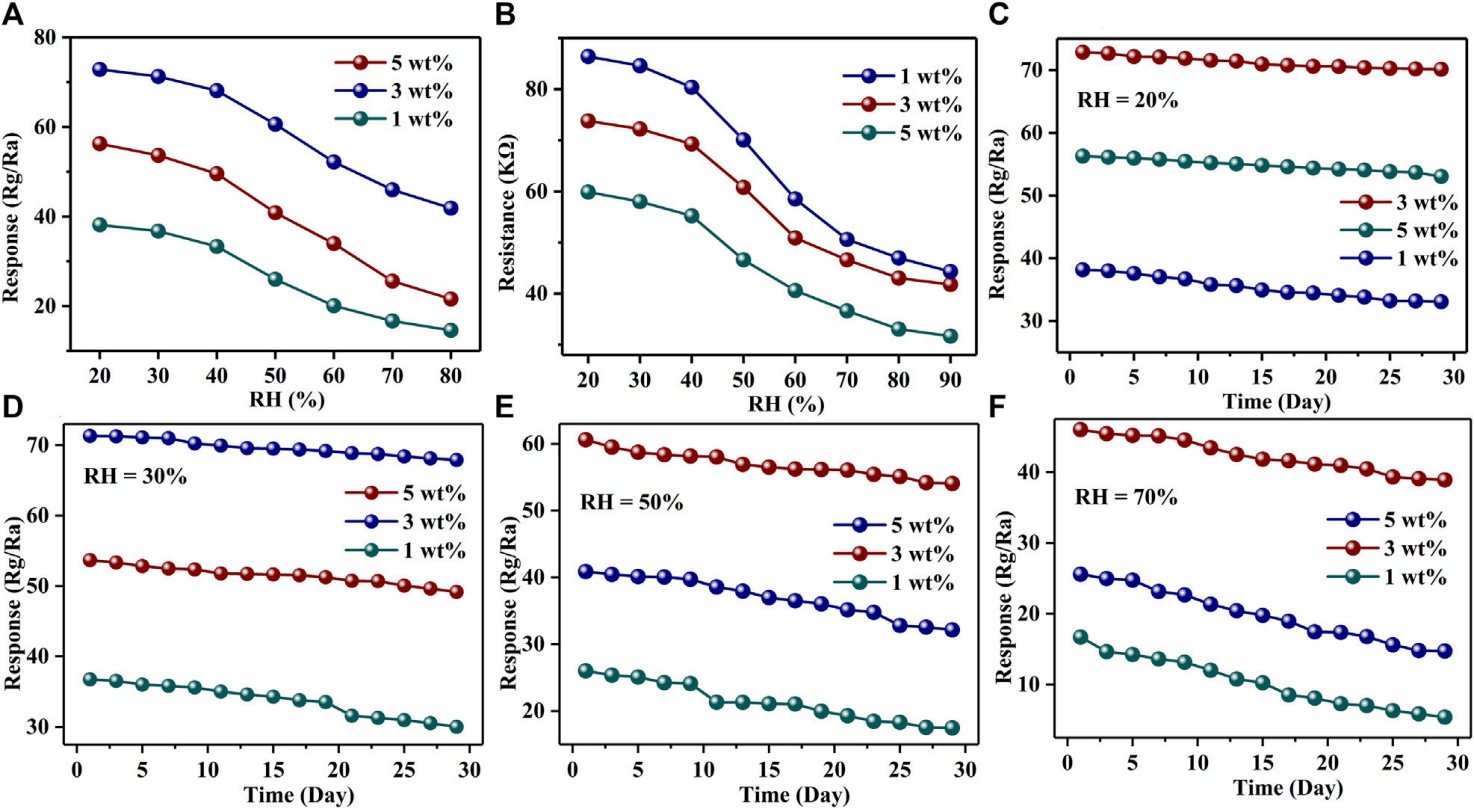
**(A)** The response changing of Au-PrFeO_3_ with RH; **(B)** The resistance changing of Au-PrFeO_3_ with RH; **(C–F)** The long-term stability of responses of Au-PrFeO_3_ under different RH.

The relative humidity (RH) in the environment is also a factor that cannot be ignored in the application of gas sensors. Figures 7A and Supplementary Figure S7 show the responses of Au–PrFeO_3_ to 1 ppm H_2_S with varying RH. It can be seen that the response decreased with increasing RH: before 50% RH, the response was little affected by it; however, above 50% RH, the responses decreased sharply. This means that the gas sensor in this study can be used in a low-RH environment without considering the influence of RH. This will greatly expand its practical application field. Figures 7B and Supplementary Figure S8 show the resistance change of Au–PrFeO3 with RH. For Au–PrFeO_3_, the resistance decreased with RH, but the proportion of decrease differed. In the 20–90% RH range, the proportion of decreases were 53.21% (0 wt% Au), 48.6% (1 wt% Au), 41.8% (3 wt% Au), and 47.09% (5 wt% Au). Thus, the resistance of 3 wt% Au–PrFeO_3_ presented the highest RH adaptability.

Long-term stability is another important property for gas-sensing materials. The higher the long-term stability, the longer the replacement cycle of the gas-sensing material and, so, the more economic and energy advantages it has. Figures 7C–F show the long-term stability of Au–PrFeO_3_ under different RH over 30 days. The experimental data were obtained every 2 days. It can be seen that all of the responses decreased slightly with time, but the proportion of decrease was lowest when the sensor was kept at under 20% RH. The proportions of decrease when the sensor was kept at under 20% RH were 34.9% (0 wt% Au), 13.3% (1 wt% Au), 3.7% (3 wt% Au), and 5.7% (5 wt% Au). It can be seen that the long-term stability of 3 wt% Au–PrFeO_3_ was more than 9 times that of pure PrFeO_3_. Therefore,Au-doped PrFeO_3_ demonstrated advantages, in terms of long-term stability. Other types of sensors, such as MOX (Pashami et al., 2012; Li et al., 2021) and MWCNT (Pistone et al., 2013; Barthwal and Singh, 2020) have been shown to have good stability under high RH environments. However, MOS, MOX, and MWCNT gas sensors are affected by RH in practical applications; therefore, improving their RH adaptability is a keyway to broaden their application field.

## Sensing mechanism analysis

**FIGURE 8:**
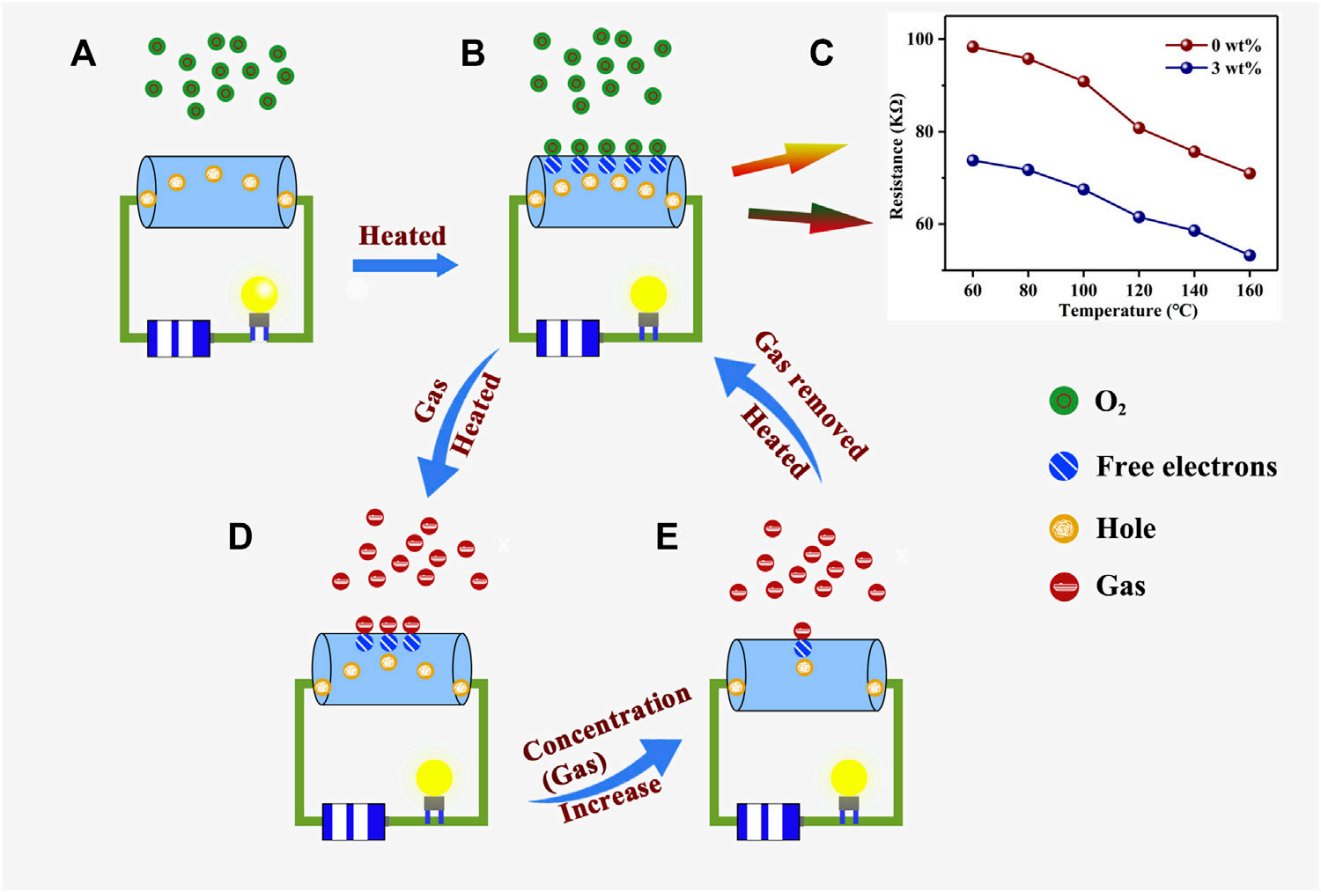
The reaction mechanism of the whole experiment in this work. **(A)** The hole is the main carriers in Au-PrFeO_3_; **(B)** At high operating temperatures, the oxygen molecules capture electrons from the surface of the Au-PrFeO_3_; **(C)** The resistance changing of Au-PrFeO_3_ at any operating temperature; **(D)** At high operating temperatures, the H_2_S gas molecules react with oxygen ions on the surface of the Au-PrFeO_3_; **(E)** At high operating temperatures, the response increase with the concentration of H_2_S gas molecules.

Figure 8 shows the reaction mechanism for the experiment conducted in this work. At room temperature (20°C), for a p-type semiconductor, the main carrier of Au–PrFeO_3_ is the hole ( $h^{•}$ ; Figure 8A). According to Kröger–Vink defect notation, the holes are mainly produced by the ionization of [ $V_{Pr}^{x}$ ], the reaction may like this:

$$\begin{array}{c} V_{Pr}^{x}\to V_{Pr}^{\prime \;\prime \;\prime }\mathrm{+3}h^{•} \end{array}$$ (4)

Before Au doping, few oxygen molecules capture the free electrons from the material, resulting in the formation of oxygen ions on the material’s surface and few holes are created in this process at the same time. As the work function of Au is larger than that of PrFeO_3_, electrons will transfer from PrFeO_3_ to the surrounding Au nanoparticles after Au doping, resulting in an increase in the number of holes in PrFeO_3_ (Figure 8B). This reaction may look like:

$$\begin{array}{c} O_{2}+\;e^{-}\;\to \;O_{2}^{-}\left(ads\right)+\;h^{+} \end{array}$$ (5)

$$\begin{array}{c} O_{2}^{-}\left(ads\right)+\;e^{-}\;\to \;2O^{-}\left(ads\right)+\;h^{+} \end{array}$$ (6)

where *ads* denote the state where oxygen is adsorbed on the material surface.

In order to verify this theoretical assumption, the resistances of pure PrFeO_3_ and 3 wt% Au–PrFeO_3_ were tested, and the results are shown in Figure 8C. It can be seen that the resistance of 3 wt% Au–PrFeO_3_ was lower than that of PrFeO_3_ at any operating temperature, consistent with the above theoretical assumption.

When the H_2_S gas molecule is introduced, it will be adsorbed onto the surface of the PrFeO_3_ to react with the oxygen ions (Figure 8D). The adsorption and desorption on the surface of Au–PrFeO_3_ of H_2_S gas molecules exist simultaneously. The rates of adsorption and desorption increase with the operating temperature, where the rate of adsorption is greater than the rate of desorption before the operating temperature reaches the optimum temperature. Therefore, the count of adsorbed H_2_S molecules on the surface of the material increases, and the reaction between H_2_S molecules and oxygen ions is more intense, resulting in an increased response. When the operating temperature exceeds the optimum temperature, the rate of adsorption of Au–PrFeO_3_ with respect to H_2_S molecules is lower than the rate of desorption and the intensity of the reaction between H_2_S molecule and oxygen ions is reduced, causing the response to decrease. Furthermore, at the optimum temperature, as the concentration of H_2_S gas molecule increases, the number of H_2_S molecules adsorbed on the surface of the Au–PrFeO_3_ will increase, causing the response to increase (Figure 8E). However, the number of free electrons on the surface of the Au–PrFeO_3_ is not infinite, and the energy required to make an electronic transition within Au–PrFeO_3_ is also increasing. Therefore, the response ( $R_{g}/R_{a}$ ) increases with the concentration of H_2_S gas molecules, but the rate of increase declines. In addition, when the free electron is released from oxygen ions adsorbed onto the Au–PrFeO_3_, the PrFeO_3_ in the depletion layer width narrowing caused by Au, resulting in a greater resistance change.

The reaction between H_2_S molecules and oxygen ions may as follows:

$$\begin{array}{c} 2H_{2}S+\;3O_{2}^{-}\;\left(ad\right)\;\to 2SO_{2}+\;2H_{2}O+3e^{-} \end{array}$$ (7)

$$\begin{array}{c} e^{-}+h^{+}\;\to null \end{array}$$ (8)

Additionally, it is well-known that the oxygen in air can be adsorbed onto the surface of semiconductor metal oxides to become oxygen ions, for which Au is a good catalyst. In this work, with the assistance of Au, oxygen molecules can more easily be adsorbed onto the surface of PrFeO_3_, due to the spillover effect (Kung et al., 2007; Wang et al., 2013). For this reason, more oxygen gets adsorbed and captures free electrons to form oxygen ionic species (Liu et al., 2011). This process increases both the quantity of adsorbed oxygen and the molecule–ion conversion rate, resulting in a high gas response (Wang et al., 2012).

## Application in the detection of H_2_S

**FIGURE 9:**
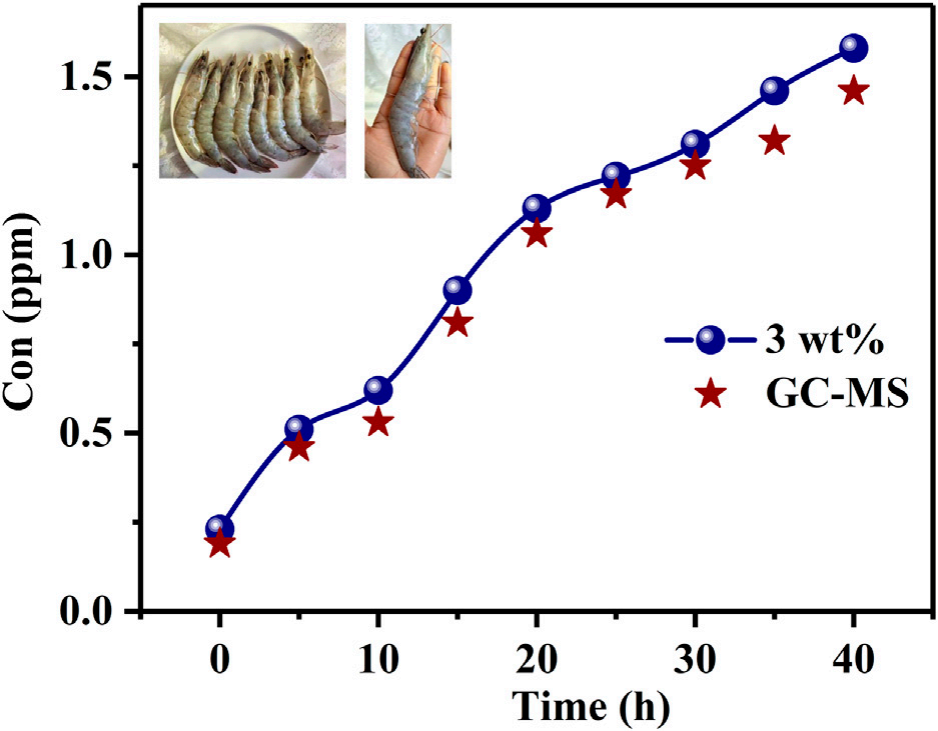
The H_2_S concentration around shrimp with time is detected by gas sensor and GC-MS method.

Accurately and quickly assessing whether meat and seafood have decomposed or not is very important. H_2_S is thought to be one of the most important gases released in the decomposition of food. The H_2_S concentration around shrimp with time was detected using the gas sensor designed in this study and GC-MS, as shown in Figure 9. Eight shrimps were placed in the experimental apparatus, each about 10–16 cm in length. It can be seen that the concentration of H_2_S increased with death-time, and the concentration of H_2_S measured by the designed gas sensor was greater than that measured by GC-MS at any time, which indicates that there were other gases in the surrounding air of the shrimp, which can have an effect on the gas sensor; however, this effect was very small. By comparing the H_2_S concentrations measured by the two methods, the error was within 10%. The results are provided in Table 3.

**TABLE 3:** The concentration of H_2_S obtained by gas sensor and GC-MS method.

Time(h)Method	0	5	10	15	20	25	30	35	40
Gas sensor	0.23	0.51	0.62	0.9	1.13	1.22	1.31	1.46	1.58
GC-MS	0.20	0.46	0.53	0.81	1.06	1.17	1.25	1.32	1.46

## Conclusion

In this study, Au-modified PrFeO_3_ was synthesized using an electrospinning method. It has a large specific surface area and high porosity, which improved the response to a certain extent. Our experimental results demonstrated that the optimum Au doping content was 3 wt%. The response of 3 wt% Au–PrFeO_3_ to H_2_S was more than 10 times higher, and its long-term stability was more than 9 times that of pure PrFeO_3_. Moreover, the response–recovery time of 3 wt% Au–PrFeO_3_ was more than 10 s shorter than that of the pure PrFeO_3_. In addition, the doping of Au, as a catalyst, greatly improved the RH adaptability and selectivity of the material. Finally, the designed Au–PrFeO_3_ was shown to be very accurate for detecting the concentration of H_2_S gas in the air around shrimp, with an error of less than 15%, when compared to the results obtained by GC-MS. Our experimental results fully demonstrate the advantages of noble metal doping in improving the performance of gas-sensing materials and the great potential of Au–PrFeO_3_ in H_2_S detection.

## Data Availability

The raw data supporting the conclusions of this article will be made available by the authors, without undue reservation.

## References

[B1] Addabbo T. Bertocci F. Fort A. Gregorkiewitz M. Mugnaini M. Spinicci R. (2015). Gas sensing properties and modeling of YCoO3 based perovskite materials. Sensors Actuators B Chem. 221, 1137–1155. 10.1016/j.snb.2015.07.079

[B2] Ahmed A. M. Mehaney A. Elsayed H. A. (2021). Detection of toluene traces in exhaled breath by using a 1D PC as a biomarker for lung cancer diagnosis. Eur. Phys. J. Plus 136, 626. 10.1140/epjp/s13360-021-01621-7

[B3] Akamatsu T. Itoh T. Tsuruta A. Masuda Y. (2021). CH3SH and H2S sensing properties of V2O5/WO3/TiO2 gas sensor. Chemosensors 9, 113. 10.3390/chemosensors9050113

[B4] Alrowaili Z. A. Elsayed H. A. Ahmed A. M. Taha T. A. Mehaney A. (2022). Simple, efficient and accurate method toward the monitoring of ethyl butanoate traces. Opt. Quantum Electron. 54, 126. 10.1007/s11082-021-03497-4 35095173 PMC8783197

[B5] Ameen A. A. Elsayed H. Aly A. H. (2021). Towards a highly efficient air purifier using annular photonic crystals in UV regimes. RSC Adv. 11, 14915–14921. 10.1039/d1ra00991e 35424060 PMC8698252

[B6] Bae G. Kim M. Lee A. Ji S. Jang M. Yim S. (2022). Nanometric lamination of zinc oxide nanofilms with gold nanoparticles for self-perceived periodontal disease sensors. Compos. Part B Eng. 230, 109490. 10.1016/j.compositesb.2021.109490

[B7] Balamurugan C. Lee D.-W. (2015). Perovskite hexagonal YMnO3 nanopowder as p-type semiconductor gas sensor for H2S detection. Sensors Actuators B Chem. 221, 857–866. 10.1016/j.snb.2015.07.018

[B8] Barthwal S. Singh N. B. (2020). Urea detection by ZnO-MWCNT nanocomposite sensor. Mater. Today Proc. 29, 749–752. 10.1016/j.matpr.2020.04.514

[B9] Behi S. Bohli N. Casanova-Cháfer J. Llobet E. Abdelghani A. (2020). Metal oxide nanoparticle-decorated few layer Graphene nanoflake chemoresistors for the detection of aromatic volatile organic compounds. Sensors 20, 3413. 10.3390/s20123413 PMC7349069 32560414

[B10] Behi S. Casanova-Chafer J. González E. Bohli N. Llobet E. Abdelghani A. (2022). Metal loaded nano-carbon gas sensor array for pollutant detection. Nanotechnology 33, 195501. 10.1088/1361-6528/ac4e43 35073524

[B11] Cerdà J. Arbiol J. Dezanneau G. Díaz R. Morante J. R. (2002). Perovskite-type BaSnO3 powders for high temperature gas sensor applications. Sensors Actuators B Chem. 84, 21–25. 10.1016/S0925-4005(02)00005-9

[B12] Chen L. Luque R. Li Y. (2017). Controllable design of tunable nanostructures inside metal–organic frameworks. Chem. Soc. Rev. 46, 4614–4630. 10.1039/C6CS00537C 28516998

[B13] Deng X. Sang S. Li P. Li G. Gao F. Sun Y. (2013). Preparation, characterization, and mechanistic understanding of Pd-decorated ZnO nanowires for ethanol sensing. J. Nanomater. 2013, 1–8. 10.1155/2013/297676

[B14] Ethiraj J. Bonino F. Lamberti C. Bordiga S. (2015). H2S interaction with HKUST-1 and ZIF-8 MOFs: A multitechnique study. Microporous Mesoporous Mat. 207, 90–94. 10.1016/j.micromeso.2014.12.034

[B15] Fan J. Liu P. Chen X. Zhou H. Fu S. Wu W. (2019). Carbon nanotubes-CuO/SnO2 based gas sensor for detecting H2S in low concentration. Nanotechnology 30, 475501. 10.1088/1361-6528/ab3cb3 31426042

[B16] Gao H. Wei D. Lin P. Liu C. Sun P. Shimanoe K. (2017). The design of excellent xylene gas sensor using Sn-doped NiO hierarchical nanostructure. Sensors Actuators B Chem. 253, 1152–1162. 10.1016/j.snb.2017.06.177

[B17] Guo L. Xie N. Wang C. Kou X. Ding M. Zhang H. (2018). Enhanced hydrogen sulfide sensing properties of Pt-functionalized α-Fe2O3 nanowires prepared by one-step electrospinning. Sensors Actuators B Chem. 255, 1015–1023. 10.1016/j.snb.2017.07.055

[B18] Han T. Ma S. Y. Xu X. L. Xu X. H. Pei S. T. Tie Y. (2020). Rough SmFeO3 nanofibers as an optimization ethylene glycol gas sensor prepared by electrospinning. Mat. Lett. 268, 127575. 10.1016/j.matlet.2020.127575

[B19] Hosoya Y. Itagaki Y. Aono H. Sadaoka Y. (2005). Ozone detection in air using SmFeO3 gas sensor. Sensors Actuators B Chem. 108, 198–201. 10.1016/j.snb.2004.10.059

[B20] Hsu K.-C. Fang T.-H. Hsiao Y.-J. Li Z.-J. (2021). Rapid detection of low concentrations of H2S using CuO-doped ZnO nanofibers. J. Alloys Compd. 852, 157014. 10.1016/j.jallcom.2020.157014

[B21] Huang H. T. Zhang W. L. Zhang X. D. Guo X. (2018). NO2 sensing properties of SmFeO3 porous hollow microspheres. Sensors Actuators B Chem. 265, 443–451. 10.1016/j.snb.2018.03.073

[B22] Jaouali I. Hamrouni H. Moussa N. Nsib M. F. Centeno M. A. Bonavita A. (2018). LaFeO3 ceramics as selective oxygen sensors at mild temperature. Ceram. Int. 44, 4183–4189. 10.1016/j.ceramint.2017.11.221

[B23] Kumar V. Majhi S. M. Kim K.-H. Kim H. W. Kwon E. E. (2021). Advances in In2O3-based materials for the development of hydrogen sulfide sensors. Chem. Eng. J. 404, 126472. 10.1016/j.cej.2020.126472

[B24] Kung M. C. Davis R. J. Kung H. H. (2007). Understanding Au-catalyzed low-temperature CO oxidation. J. Phys. Chem. C 111, 11767–11775. 10.1021/jp072102i

[B25] Li W. Huang L. Wang T. Hao X. Wang B. Lu Q. (2021). Based Nafion gas sensor utilizing Pt-MOx (MOx = SnO2, In2O3, CuO) sensing electrode for CH3OH detection at room temperature in FCVs. Sensors Actuators B Chem. 346, 130543. 10.1016/j.snb.2021.130543

[B26] Li X. Hu J. Ban J. He S. Zheng N. Shao G. (2022a). Mechanism of enhanced H2S sensor ability based on emerging Li0.5La0.5TiO3-SnO2 core-shell structure. Sensors Actuators B Chem. 352, 131054. 10.1016/j.snb.2021.131054

[B27] Li X. Yang H. Wang X. Qin Z. Hu X. Wang X. (2022b). Exposed edges of porous ultrathin WO3 nanosheets determined High-performance sensing for hydrogen sulfide. Appl. Surf. Sci. 571, 151327. 10.1016/j.apsusc.2021.151327

[B28] Lim K. Jo Y. M. Kim S. Yoon J. W. Jeong S. Y. Kim J. S. (2021). Selective dual detection of hydrogen sulfide and methyl mercaptan using CuO/CuFe2O4 nanopattern chemiresistors. Sensors Actuators B Chem. 348, 130665. 10.1016/j.snb.2021.130665

[B29] Liu X. Zhang J. Wang L. Yang T. Guo X. Wu S. (2011). 3D hierarchically porous ZnO structures and their functionalization by Au nanoparticles for gas sensors. J. Mat. Chem. 21, 349–356. 10.1039/c0jm01800g

[B30] Liu B. Zhang L. Luo Y. Gao L. Duan G. (2021). The dehydrogenation of HS bond into sulfur species on supported Pd single atoms allows highly selective and sensitive hydrogen sulfide detection. Small 17, 2105643. 10.1002/smll.202105643 34716747

[B31] Lopez J. D. Keley M. Dante A. Werneck M. M. (2021). Optical fiber sensor coated with copper and iron oxide nanoparticles for hydrogen sulfide sensing. Opt. Fiber Technol. 67, 102731. 10.1016/j.yofte.2021.102731

[B32] Ma L. Ma S. Y. Shen X. F. Wang T. T. Jiang X. H. Chen Q. (2018). PrFeO3 hollow nanofibers as a highly efficient gas sensor for acetone detection. Sensors Actuators B Chem. 255, 2546–2554. 10.1016/j.snb.2017.09.060

[B33] Ma Z. Yang K. Xiao C. Jia L. (2021). C-doped LaFeO3 porous nanostructures for highly selective detection of formaldehyde. Sensors Actuators B Chem. 347, 130550. 10.1016/j.snb.2021.130550

[B34] Mehaney A. Alrowaili Z. A. Elsayed H. A. Taha T. A. Ahmed A. M. (2021). Theoretical investigations of Tamm plasmon resonance for monitoring of isoprene traces in the exhaled breath: Towards chronic liver fibrosis disease biomarkers. Phys. Lett. A 413, 127610. 10.1016/j.physleta.2021.127610

[B35] Mehta S. S. Nadargi D. Y. Tamboli M. S. Alshahrani T. Minnam Reddy V. R. Kim E. S. (2021). RGO/WO3 hierarchical architectures for improved H2S sensing and highly efficient solar-driving photo-degradation of RhB dye. Sci. Rep. 11, 5023. 10.1038/s41598-021-84416-1 33658543 PMC7930058

[B36] Pan W. Zhang Y. Yu S. Liu X. Zhang D. (2021). Hydrogen sulfide gas sensing properties of metal organic framework-derived α-Fe2O3 hollow nanospheres decorated with MoSe2 nanoflowers. Sensors Actuators B Chem. 344, 130221. 10.1016/j.snb.2021.130221

[B37] Pashami S. Lilienthal A. Trincavelli M. (2012). Detecting changes of a distant gas source with an array of MOX gas sensors. Sensors 12, 16404–16419. 10.3390/s121216404 23443385 PMC3571789

[B38] Peng F. Yu W. Lu Y. Sun Y. Fu X. Hao J. M. (2020). Enhancement of low-temperature gas-sensing performance using substoichiometric WO_3–x_ modified with CuO. ACS Appl. Mat. Interfaces 12, 41230–41238. 10.1021/acsami.0c09213 32804471

[B39] Pistone A. Piperno A. Iannazzo D. Donato N. Latino M. Spadaro D. (2013). Fe3O4–MWCNTPhCOOH composites for ammonia resistive sensors. Sensors Actuators B Chem. 186, 333–342. 10.1016/j.snb.2013.06.027

[B40] Priya A. K. Suresh R. Kumar P. S. Rajendran S. Vo D.-V. N. Soto-Moscoso M. (2021). A review on recent advancements in photocatalytic remediation for harmful inorganic and organic gases. Chemosphere 284, 131344. 10.1016/j.chemosphere.2021.131344 34225112

[B41] Senapati M. Sahu P. P. (2020). Meat quality assessment using Au patch electrode Ag-SnO2/SiO2/Si MIS capacitive gas sensor at room temperature. Food Chem. 324, 126893. 10.1016/j.foodchem.2020.126893 32344336

[B42] Sheng H. Ma S. Han T. Yun P. Yang T. Ren J. (2022). A highly sensitivity and anti-humidity gas sensor for ethanol detection with NdFeO3 nano-coral granules. Vacuum 195, 110642. 10.1016/j.vacuum.2021.110642

[B43] Song P. Zhang H. Han D. Li J. Yang Z. Wang Q. (2014). Preparation of biomorphic porous LaFeO3 by sorghum straw biotemplate method and its acetone sensing properties. Sensors Actuators B Chem. 196, 140–146. 10.1016/j.snb.2014.02.006

[B44] Song Y. Zhang Y. Ma M. Ren J. Liu C. Tan J. (2020). Visible light-assisted formaldehyde sensor based on HoFeO3 nanoparticles with sub-ppm detection limit. Ceram. Int. 46, 16337–16344. 10.1016/j.ceramint.2020.03.191

[B45] Thamri A. Baccar H. Casanova-Chafer J. Mejri M. B. Llobet E. Abdelghani A. (2021). Thiol-amine functionalized decorated carbon nanotubes for biomarker gases detection. Chemosensors 9, 87. 10.3390/chemosensors9050087

[B46] Tomoda M. Okano S. Itagaki Y. Aono H. Sadaoka Y. (2004). Air quality prediction by using semiconducting gas sensor with newly fabricated SmFeO3 film. Sensors Actuators B Chem. 97, 190–197. 10.1016/j.snb.2003.08.013

[B47] Wang L. Lou Z. Fei T. Zhang T. (2012). Templating synthesis of ZnO hollow nanospheres loaded with Au nanoparticles and their enhanced gas sensing properties. J. Mat. Chem. 22, 4767–4771. 10.1039/c2jm15342d

[B48] Wang L. Dou H. Lou Z. Zhang T. (2013). Encapsuled nanoreactors (Au@SnO2): a new sensing material for chemical sensors. Nanoscale 5, 2686. 10.1039/c2nr33088a 23295974

[B49] Wang Q. Huang J. Zhou J. Liu Z. Geng Y. Liang Z. (2018). Different nanostructured tungsten oxides synthesized by facile solvothermal route for chlorine gas sensing. Sensors Actuators B Chem. 275, 306–311. 10.1016/j.snb.2018.08.047

[B50] Wang X. Lu J. Han W. Yang J. Jiang B. Sun Y. (2021). Co-PBA MOF-derived hierarchical hollow Co3O4@NiO microcubes functionalized with Pt for superior H2S sensing. Sensors Actuators B Chem. 342, 130028. 10.1016/j.snb.2021.130028

[B51] Wu X. Xiong S. Gong Y. Gong Y. Wu W. Mao Z. (2019). MOF-SMO hybrids as a H2S sensor with superior sensitivity and selectivity. Sensors Actuators B Chem. 292, 32–39. 10.1016/j.snb.2019.04.076

[B52] Xiangfeng C. Siciliano P. (2003). CH3SH-sensing characteristics of LaFeO3 thick-film prepared by co-precipitation method. Sensors Actuators B Chem. 94, 197–200. 10.1016/S0925-4005(03)00340-X

[B53] Yaegaki K. Sanada K. (1992). Volatile sulfur compounds in mouth air from clinically healthy subjects and patients with periodontal disease. J. Periodontal Res. 27, 233–238. 10.1111/j.1600-0765.1992.tb01673.x 1640345

[B54] Yao X. Zhao J. Liu J. Wang F. Wu L. Meng F. (2022). H2S sensing material Pt-WO3 nanorods with excellent comprehensive performance. J. Alloys Compd. 900, 163398. 10.1016/j.jallcom.2021.163398

[B55] Yin Y. Shen Y. Zhou P. Lu R. Li A. Zhao S. (2020). Fabrication, characterization and n-propanol sensing properties of perovskite-type ZnSnO3 nanospheres based gas sensor. Appl. Surf. Sci. 509, 145335. 10.1016/j.apsusc.2020.145335

[B56] Zhang Y. Zhang X. Guo C. Xu Y. Cheng X. Zhang F. (2020). Novel two-dimensional WO 3/Bi 2 W 2 O 9 nanocomposites for rapid H 2 S detection at low temperatures. ACS Appl. Mat. Interfaces 12, 54946–54954. 10.1021/acsami.0c15611 33241936

[B57] Zheng X. Zhang G. Yao Z. Zheng Y. Shen L. Liu F. (2021). Engineering of crystal phase over porous MnO2 with 3D morphology for highly efficient elimination of H2S. J. Hazard. Mat. 411, 125180. 10.1016/j.jhazmat.2021.125180 33858115

[B58] Zhou Q. Xu L. Kan Z. Yang L. Chang Z. Dong B. (2022). A multi-platform sensor for selective and sensitive H2S monitoring: Three-dimensional macroporous ZnO encapsulated by MOFs with small Pt nanoparticles. J. Hazard. Mat. 426, 128075. 10.1016/j.jhazmat.2021.128075 34959212

[B59] Zuo P. Wang R. Li F. Wu F. Xu G. Niu W. (2021). A trace ppb-level electrochemical H2S sensor based on ultrathin Pt nanotubes. Talanta 233, 122539. 10.1016/j.talanta.2021.122539 34215042

