# Supplementary material for: Au modified PrFeO3 with hollow tubular structure can be efficient sensing material for H2S detection
Source: Front Bioeng Biotechnol. 2022 Aug 24;10:969870. doi: 10.3389/fbioe.2022.969870 (PMC9449130; doi:10.3389/fbioe.2022.969870)
Supplement: Supplementary file 1 [file DataSheet1.docx]

Supplementary Material

Au modified PrFeO_3_ with hollow tubular structure can be efficient sensing material for H_2_S detection

Heng Zhang^1^, Jing Xiao^1*^, Jun Chen^1^, Lian Zhang^1^, Yi Zhang^1^, Pan Jin^2,3*^

^1^ College of Physics and Electronic Engineering, Taishan University, Taian, Shandong 271000, China

^2^ Health Science Center, Yangtze University, Jingzhou 434023, Hubei, China

^3^ Collaborative Innovation Centre of Regenerative Medicine and Medical BioResource Development and Application Co-constructed by the Province and Ministry, Guangxi Medical University, Nanning 530021, Guangxi, China

*** Correspondence:**JingXiao, xiaojingzx@163.com; Pan Jin, jinpanmountain@163.com;

**Table captions**

**Table S1.** The response and recovery time of Au-PrFeO_3_ at different operating temperature.

| **T (℃)**  **Time** | **60** | **80** | **100** | **120** | **140** | **160** | **Au (wt%)** |
| --- | --- | --- | --- | --- | --- | --- | --- |
| Response | 32.11 | 35.03 | 38.26 | 40.35 | 37.36 | 33.69 | 0 |
| Recovery | 25.37 | 28.22 | 31.12 | 32.78 | 29.06 | 25.87 | 0 |
| Response | 26.33 | 29.6 | 31.03 | 33.36 | 31.14 | 27.14 | 1 |
| Recovery | 20.68 | 23.56 | 25.1 | 28.63 | 25.89 | 22.26 | 1 |
| Response | 20.53 | 22.50 | 25.91 | 28.48 | 26.96 | 23.91 | 3 |
| Recovery | 11.75 | 13.50 | 15.89 | 18.96 | 13.68 | 10.93 | 3 |
| Response | 21.34 | 25.6 | 28.03 | 31.36 | 29.14 | 24.14 | 5 |
| Recovery | 17.66 | 20.03 | 21.89 | 23.31 | 21.22 | 18.21 | 5 |

**Figure S1.**


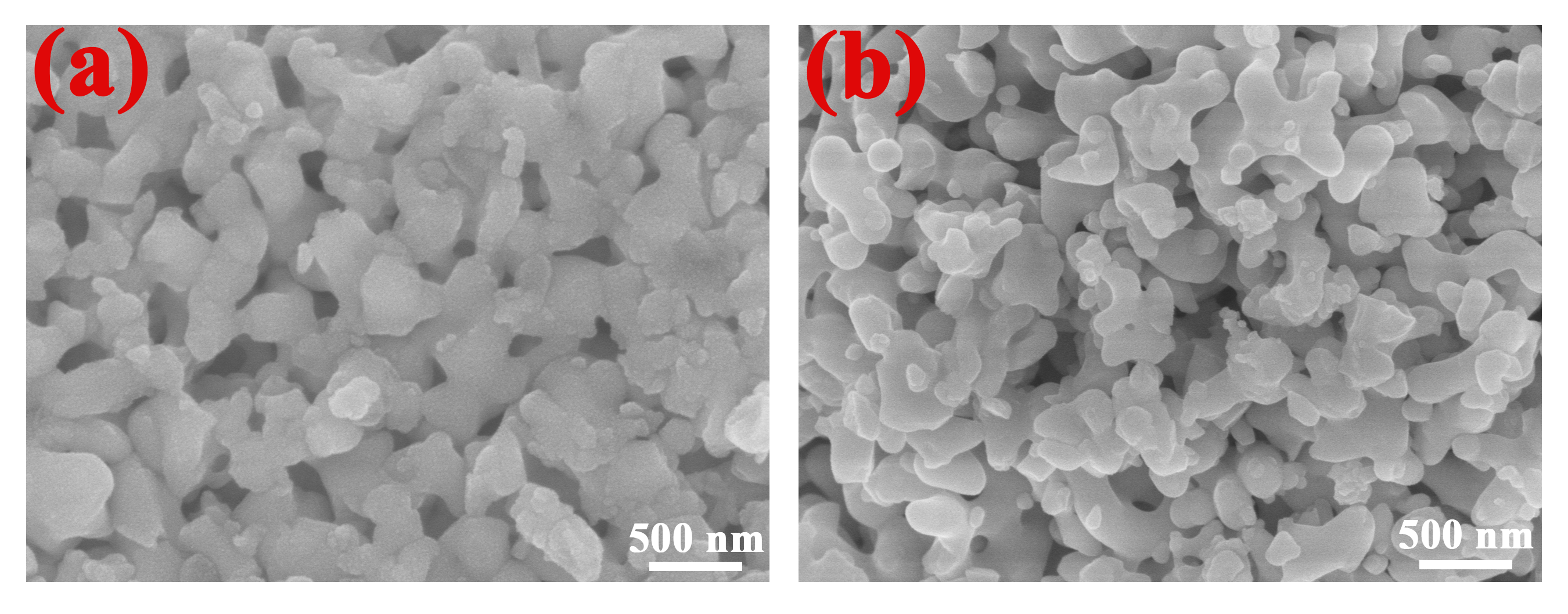


The Scanning Electron Microscope (SEM) pattern of PrFeO_3_.

**Figure S2.**

**

**

The response curves of PrFeO_3_ to 1 ppm H_2_S with the operating temperature.

**Figure S3.**





The relationship between the response of PrFeO_3_ and multiple H_2_S concentration.

**Figure S4.**





The repeatability of responses to different concentration of H_2_S gas.

**Figure S5.**





The response-recovery time of PrFeO_3_ to 1 ppm H_2_S.

**Figure S6.**

**

**

The selectivity comparison of PrFeO_3_ to 1 ppm H_2_S and several other common gases.

**Figure S7.**





The responses of PrFeO_3_ to 1 ppm H_2_S with RH.

**Figure S8.**





The resistance changing of PrFeO_3_ with RH.

**Figure S9.**





The long-term stability of H_2_S-response of PrFeO_3_ in 30 days.
